# Investigating the Impact of Curing System on Structure-Property Relationship of Natural Rubber Modified with Brewery By-Product and Ground Tire Rubber

**DOI:** 10.3390/polym12030545

**Published:** 2020-03-03

**Authors:** Łukasz Zedler, Xavier Colom, Javier Cañavate, Mohammad Reza Saeb, Józef T. Haponiuk, Krzysztof Formela

**Affiliations:** 1 Department of Polymer Technology, Faculty of Chemistry, Gdańsk University of Technology, Gabriela Narutowicza 11/12., 80–233 Gdańsk, Poland; lukasz.zedler@pg.edu.pl (Ł.Z.); jozef.haponiuk@pg.edu.pl (J.T.H.); 2 Department of Chemical Engineering, Universitat Politècnica de Catalunya Barcelona Tech, Carrer de Colom, 1, 08222 Terrassa, Barcelona, Spain; francisco.javier.canavate@upc.edu; 3 Department of Resin and Additives, Institute for Color Science and Technology, P.O. Box, 16765-654 Tehran, Iran; saeb-mr@icrc.ac.ir

**Keywords:** biocomposites, natural rubber, recycling, brewers’ spent grain, ground tire rubber, hybrid filler, structure-property relationships

## Abstract

The application of wastes as a filler/reinforcement phase in polymers is a new strategy to modify the performance properties and reduce the price of biocomposites. The use of these fillers, coming from agricultural waste (cellulose/lignocellulose-based fillers) and waste rubbers, constitutes a method for the management of post-consumer waste. In this paper, highly-filled biocomposites based on natural rubber (NR) and ground tire rubber (GTR)/brewers’ spent grain (BSG) hybrid reinforcements, were prepared using two different curing systems: (i) sulfur-based and (ii) dicumyl peroxide (DCP). The influence of the amount of fillers (in 100/0, 50/50, and 0/100 ratios in parts per hundred of rubber) and type of curing system on the final properties of biocomposites was evaluated by the oscillating disc rheometer, Fourier-transform infrared spectroscopy, thermogravimetric analysis, scanning electron microscopy, swelling behavior, tensile testing, and impedance tube measurements. The results show, that the scorch time and the optimum curing time values of sulfur cured biocomposites are affected by the change of the hybrid filler ratio while using the DCP curing system, and the obtained values do not show significant variations. The results conclude that the biocomposites cured with sulfur have better physico-mechanical and acoustic absorption, and that the type of curing system does not influence their thermal stability. The overall analysis indicates that the difference in final properties of highly filled biocomposites cured with two different systems is mainly affected by the: (i) cross-linking efficiency, (ii) partial absorption and reactions between fillers and used additives, and (iii) affinity of additives to applied fillers.

## 1. Introduction

Growing global consumerism is one of the reasons for the accumulation of a significant amount of post-production, post-consumer, and agricultural waste. This phenomenon is a threat to society and the environment, which forces the state authorities, manufacturing companies, and scientists to develop methods for their management.

The post-utilization problem and the need to replace expensive compounds in a multicomponent product can be solved by the use of the wastes as a filler/reinforcement in polymers, preferably if it comes accompanied with new unique performance properties or if it, at least, leads to lowering the final costs of the obtained materials. Over the past two decades, cellulose/lignocellulose-based fillers (peanut shell, rice husk, kenaf fiber, bamboo fiber, etc.) have gained importance due to their biodegradability, renewable character, abundance, low density, and cost [1,2,3,4]. The bio-fillers with these features can be used to partially or completely replace commonly applied fillers in the rubber industry such as carbon black (petrochemical-based product) and silica, which are non-degradable and consume high amounts of energy during production. These aspects motivate scientists to study the incorporation of bio-based materials as modifiers of natural rubber and synthetic elastomers, obtaining biocomposites [5,6,7,8]. The final properties of composite material, including good strength and dimensional stability, mainly depend on the interfacial interactions developed between the components [9,10,11]. The issue in developing good interactions and compatibility is related to the polar and hydrophilic nature of the bio-filler, contrasting to the non-polar and rather hydrophobic rubber [12]. The problem can be solved through the chemical surface modification of the matrix and/or bio-filler, and/or through the improvement of the miscibility and compatibility between components of the biocomposites by other methods [13,14,15,16].

A similar approach can be applied to the incorporation of ground tire rubber (GTR) into a raw rubber matrix [17,18,19,20], which also reduces the cost of the final product. Applying this concept can prevent the problem of the increasing amount of waste rubber, which presents as an added difficulty the existence of a three-dimensional cross-linked structure that makes it more difficult for further processing and recycling [21].

Taking into account the advantages of the use of waste fillers (cellulose/lignocellulose-based fillers and GTR), a new strategy to improve the performance properties of biocomposites was considered in this study. Instead of applying the fillers individually, the mixture of chosen fillers can be introduced into the system The application of hybrid fillers [22,23,24] may cause the occurrence of synergistic effects, providing superior results in terms of performance than the sum of the effects for the separate filler [25,26].

However, not only modification, ratio, and type of rubber matrix and fillers have a significant influence on the performance properties of rubber biocomposites. In the fabrication of elastomers, vulcanization can be used to bond the fillers to the matrix, obtaining an improvement of the properties due to a good integration of the disperse particles into the whole material. The improvement of the properties, due to the addition of a filler into rubber matrix, may result from the physical and chemical interactions in filler-filler and filler-matrix systems [27]. For example, the simple addition of bis(triethoxysilylpropyl)tetrasulfide (TESPT) into the rubber compound results in obtaining a chemical bonding between filler and matrix [28,29].

Fillers commonly applied for the rubber products can be divided into three groups: non-reinforcing, semi-reinforcing, and reinforcing. In general, fillers can be segregated into those groups depending on their specific activity, which can be determined, as mentioned before, by the physical and chemical nature of the surface, either of the filler as well as a its interfacial interactions with matrix. Sae-Oui et al. [30] investigated if a rice husk (low and high carbon content) can be used as a filler for natural rubber. The results were compared with several commercially available fillers commonly used in the rubber industry (talc, clay, calcium carbonate, silica, and carbon black) in four ratios (15, 30, 45, and 60 phr). The results were compared with a reference sample (unfilled natural rubber). All the products were vulcanized using a sulfur curing system. According to the results with the increasing loading of the fillers the hardness and tear strength increase (especially for carbon black and silica), but the tensile strength and elongation at break decrease, particularly for naturally derived filler. The authors explained that the deterioration of the properties was caused by a weak interaction between rice husk and the matrix, as well as the porous structure of the filler. The better properties (than reference sample) were achieved by applying talc, clay, silica, and carbon black (<30 phr). In the case of the last two fillers, the properties can be partially attributed to the high surface area. However, talc and clay have a significantly lower value of that property (what translates to a higher drop of properties with higher loadings). Talc and clay are rather non-reinforcing fillers. With a low amount, it is possible that the filler enhances a phenomenon occurring for natural rubber during a tensile test, which is strain-induced crystallization. It locally amplifies the stress for macromolecular chains (shifting the crystallization process) resulting in higher stress and faster break of the structure (lower elongation at break). Those assumptions are in accordance with the presented results.

Moreover, the properties of the vulcanized products are strictly dependent on the applied curing system [31], which will have an influence on the rheological, mechanical, and physico-chemical properties [32,33,34,35]. As a result of the used curing system and processing temperatures, interactions between other components and the elastomeric matrix and dispersion will show considerable variations.

In this paper, highly-filled biocomposites based on natural rubber/brewers’ spent grain/ground tire rubber system (with different ratio of hybrid fillers) were cured with a sulfur curing system (S) and subsequently were compared with biocomposites cured with dicumyl peroxide (DCP) in terms of processing, morphology, as well as mechanical and thermal properties.

## 2. Materials and Methods

### 2.1. Materials

The characteristics of the used materials are presented and summarized in Table 1.

### 2.2. Sample Preparation

**Figure 1:**
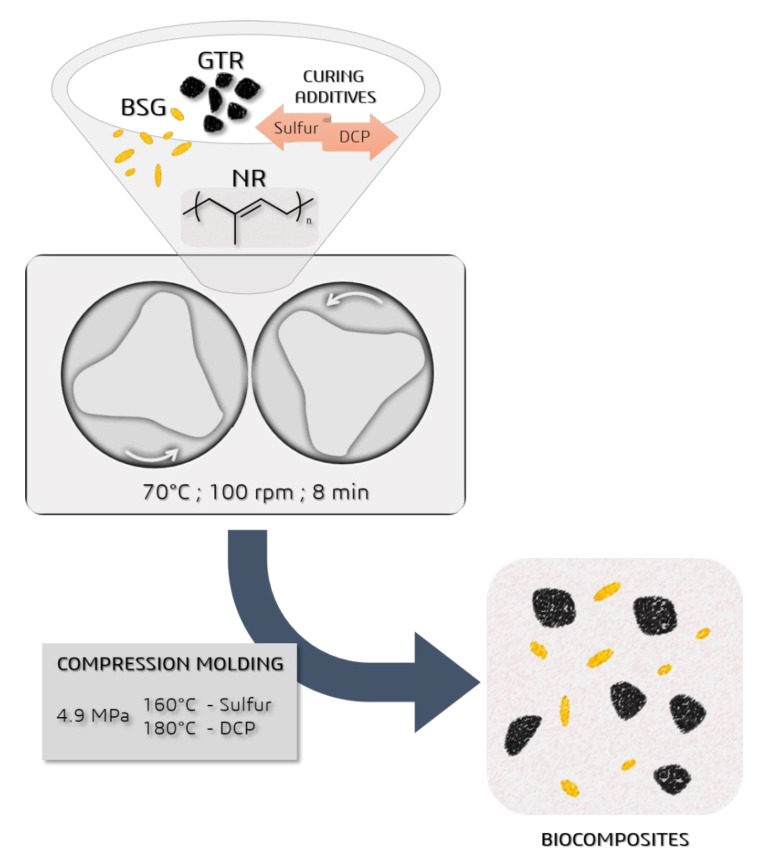
Sample preparation procedure.

Studied biocomposites were prepared using a Brabender batch mixer model GMF 106/2 (Brabender, Duisburg, Germany), homogenized using a laboratory two roll mills from Buzuluk (Buzuluk, Komárov, Czech Republic), and subsequently compression molded into 2-mm thick samples at 160 °C and 4.9 MPa (sulfur curing system), and at 180 °C (peroxide curing system) and 4.9 MPa, according to optimal cure time determined according to ISO 3417 standard. The sulfur curing system composition was (in phr): Zinc oxide 5.0; stearic acid 3.0; TBBS 1.0; TMTD 0.25; sulfur 2.0 (samples coded with index “S”), and the peroxide curing system composition was (phr): DCP 3.0 without any additional components (samples coded with index “P”). The procedure for sample preparation is illustrated in Figure 1.

### 2.3. Measurements

**Figure 2:**
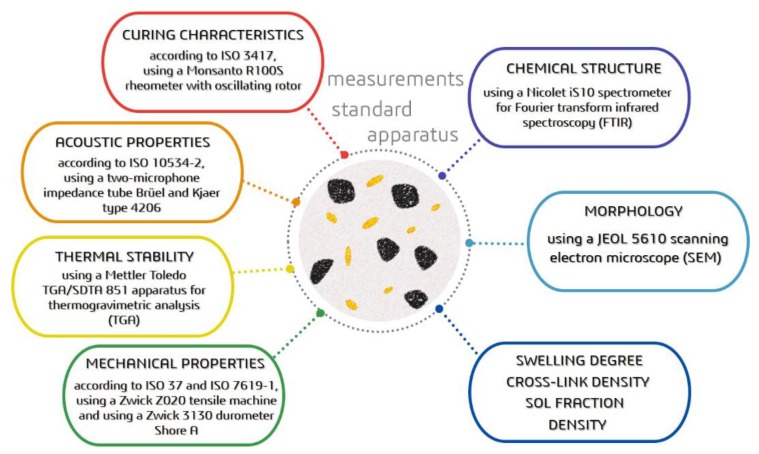
Summary of used methodology.

The methodology used in the study was summarized in Figure 2. More detailed information about the used measurement is presented in our previous work [26].

## 3. Results

### 3.1. Curing Characteristics

**Figure 3:**
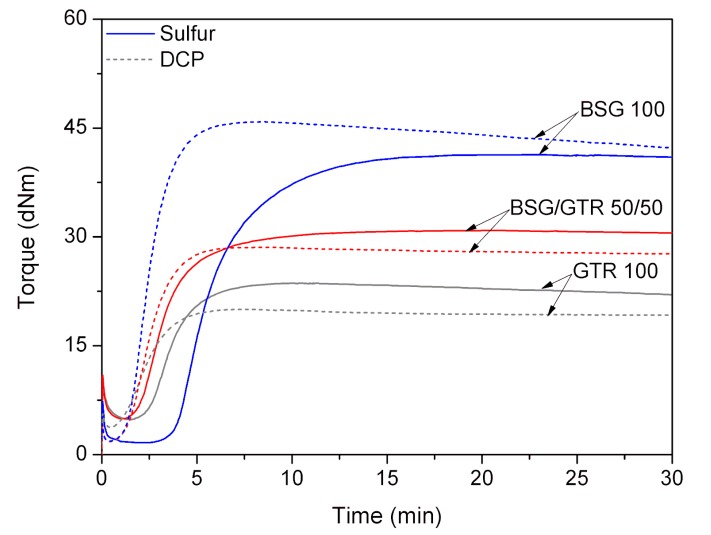
Curing curves for highly-filled NR/BSG/GTR composites.

The effect of the waste filler type (BSG, GTR or their mixture in the variable ratio) and curing agent type (sulfur curing system or DCP) on curing characteristics were analyzed and presented in Figure 3 and summarized in Table 2. It was observed that with increasing content of GTR, minimal torque (M_L_) increases for both curing systems (from 1.7 dNm—sample NR/BSG_100_^S^ to 4.8 dNm NR/GTR_100_^S^ for sulfur-based system, and from 1.9 dNm—sample NR/BSG_100_^P^ to 3.8 dNm—NR/GTR_100_^P^ for DCP, respectively). This phenomenon is due to the difference in the structure of GTR and BSG. The presence of carbon black and cross-linked structure present in GTR hinders the processing of the material, resulting in an increase of the minimal torque. On the other hand, the proteins present in BSG might act as plasticizers and facilitate the process [38], which corresponds with the obtained results. An interesting fact is that for samples BSG/GTR 50/50, curing system has a significant influence on minimal torque value. In comparison to BSG/GTR 50/50 cured with sulfur curing system, the same sample cured with DCP shows lower M_L_ (5.0 and 1.8 dNm, respectively).

The changes in maximal torque and torque increment (ΔM = M_H_ − M_L_) were noticed for both systems. As mentioned in our previous study [26], the dual nature of BSG acts in improving processing (plasticization effect of proteins present in BSG), but in later stages of vulcanization immobilizes the polymer chains. Then, the values of maximal torque, that is, when the cross-linking has already developed, are obtained with BSG only. When increasing the amount of GTR, the maximal torque values decrease in both curing systems. This phenomenon can be related to partial absorption of curing system by GTR filler and its possible curing.

Scorch time values for NR/BSG_100_^S^, NR/BSG/GTR_50/50_^S^, and NR/GTR_100_^S^ changes, respectively, as follows: 3.9, 2.0, and 2.6 min, in the sulfur system. The presence of cross-linking precursors, unreacted curing agents, and unsaturated bonds present in GTR accelerate the cross-linking reactions upon heating. During the process, the migration of accelerator occurs, leading to a decrease in scorch time [39] when adding BSG/GTR_50/50_.

The presence of cross-linking precursors and the ability of this component to curing affects the optimum cure time parameter. The presence of GTR accelerates the process of cross-linking with the sulfur-based system, as proven by the optimum cure time values (10.1, 6.4, 5.7 min) and the increment of the curing rate index (CRI) values (16.1, 22.5, 31.8 min^−1^). Scorch time, optimum cure time, and CRI of samples cured with DCP were not affected by the changes in fillers ratio. This indicates that the mechanism of the curing by peroxide includes mainly cross-linking between natural rubber chains or in any case, the action of the peroxide provides a different type of cross-linking that produces in general higher CRI values than the sulfur-based system. It may be concluded that the addition of GTR, BSG, or BSG/GTR fillers into NR matrix, followed by vulcanization in the presence of organic peroxide, does not affect cross-linking nature and chemical structure of the studied samples. This is confirmed later by the FTIR spectra presented in the next section. The curing curves are presented in Figure 3. A visible reversion can be observed in the case of NR/BSG_100_^P^, meaning that after an initial higher torque value, the torque decreases when the curing time increases. The reversion phenomenon is getting smaller with increasing content of GTR, although after 300 s, the thermal aging resistance parameter for these samples has the highest values. This may be due to the partial thermal decomposition of cellulose and hemicellulose contained in the BSG filler or evaporation of residual water, as it was proven that naturally derived fillers absorb water in biocomposites [40].

### 3.2. FTIR Analysis

**Figure 4:**
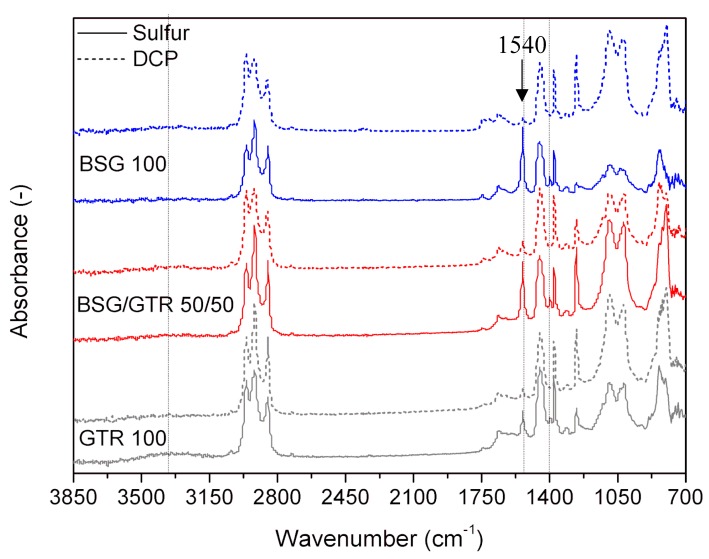
FTIR spectra for NR/BSG/GTR biocomposites.

The FTIR spectra of NR based biocomposites with different GTR/BSG ratios and different curing systems are presented in Figure 4. A small absorption band assigned to O–H stretching vibration appears at 3360 cm^−1^. This proved that material might undergo partial degradation during processing. The band is especially visible in the sample NR/GTR_100_, which means that the degradation could have taken place in the reclaimed GTR. In the case of an organic peroxide curing system, this band is not visible, probably because the peroxide can react with the present OH groups.

According to Colom et al. [41], the band at 1540 cm^−1^ is related to zinc stearate, formed during the reaction between ZnO and stearic acid. As could be expected, zinc stearate peak appears only in the samples with the sulfur curing system, because samples with DCP do not include this component.

It is worth mentioning that in the samples with 50% or 100%GTR vulcanized with DCP, there is a weak FTIR signal at 1540 cm^−1^, which is due to the first vulcanization of GTR, that has been made using the sulfur system and, therefore, also contains some ZnO and stearic acid.

The decrease of this band as a function of the amount of GTR implies that the curing process between NR and GTR is more developed than in the case of NR and BSG. Additionally, the evolution of the band at 1200 cm^−1^, assigned to the group C–O present in DCP, in BSG filler, and in the stearic acid and zinc stearate for different samples, explains how the different curing systems affecting the migration of some compounds (i.e., stearic acid, zinc stearate) from the core to the surface. ATR spectra measures the spectra on the surface of the sample. It was observed that samples with curing sulfur system with higher cross-link density show a low signal mainly in NR/GTR_100_.

This phenomenon can be related to the limitation on the migration of low molecular compounds to the surface, which is induced by the increase of cross-link density in the sample. The band at 1050 cm^−1^ assigned to C–O describes the same behavior of the band at 1200 cm^−1^, mentioned previously, in the samples cured with the sulfur system. On the other hand, the broad band around 850 cm^−1^ assigned at –C=C−H out of plane (NR) [41] is bigger in samples vulcanized with DCP than in the ones using the sulfur system. This phenomenon is related to the differences in the curing mechanism of natural rubber by DCP compared to sulfur-based system, which was comprehensively described in work [42].

### 3.3. Physico-Mechanical Properties

The physico-mechanical properties were compared and presented in Table 3. It is evident that samples cured with the sulfur-based system show better mechanical and physical properties than samples with DCP. The tensile strength changes as follows: 4.5 ± 0.1, 6.2 ± 1.4, 8.0 ± 0.4 MPa for sulfur and 2.7 ± 0.1, 3.9 ± 0.7, 3.3 ± 0.7 MPa for DCP. Similar trends are being observed for elongation at break values (475% ± 12%, 452% ± 84%, 511% ± 27% for sulfur and 221% ± 48%, 341% ± 56%, 302% ± 78% for DCP). The results showed that GTR presents a higher affinity to NR than BSG.

It is well-known that NR undergoes strain inducted crystallization [43,44,45,46]. During this phenomenon, the structure of NR partially turns from amorphous rubber into a semi-crystalline material. In this way, newly obtained highly oriented crystallites act like active filler particles or physical cross-links. The curves stress–strain (not presented in this paper) showed that the addition of BSG/GTR fillers results in the restriction of stress-induced crystallization. However, due to the presence of NR in GTR particles, the restriction is smaller for samples containing a higher amount of GTR. The higher quantity of BSG caused limitation of the polymer chains’ mobility, hence the orientation of the chains during the tensile test was also limited. The courses of stress–strain curves for sulfur cured samples and DCP cured samples are similar, only shorter than what is correlated with elongation at break values. The modulus at 100% of elongation (M100) values decreases with increasing GTR content (2.1 ± 0.1, 1.5 ± 0.2, 1.2 ± 0.1, 0.9 ± 0.2, 0.8 ± 0.1 MPa, respectively).

The hardness of the samples decreases with increasing content of GTR (66, 53, 44 Sh A for sulfur and 63, 48, 35 Sh A for DCP), which can be explained by higher elasticity of GTR than BSG. Moreover, higher hardness values for the first system prove more efficient cross-linking. The density is strictly correlated with GTR/BSG fillers ratio and samples composition. The sol fraction is described as the uncured and dissolved components of the samples washed out during the test. The increasing values of sol fraction with the increasing amount of GTR (3.38% ± 0.20%, 5.34% ± 0.20%, 7.58% ± 0.24% for sulfur, and 4.52% ± 0.03%, 6.58% ± 0.20%, 8.76% ± 0.19% for DCP) are a result of GTR structure and its reclaiming method (which was supported by bitumen). During the process scission of S–S, S–C, and C–C bonds occur causing the increase of low molecular components in its structure, which has an influence on sol fraction, swelling degree, and cross-link density of tested biocomposites. As it could be predicted, swelling degree values significantly increase with increasing GTR content (232% ± 2%, 228% ± 6%, 277% ± 4% for sulfur, and 141% ± 2%, 232% ± 4%, 307% ± 2% for DCP). Cross-link density decreases (1.80 ± 0.03, 0.87 ± 0.02, 0.62 ± 0.01 mol/cm^3^ × 10^−4^ for sulfur, and 1.89 ± 0.04, 0.85 ± 0.03, 0.53 ± 0.03 mol/cm^3^ × 10^−4^ for DCP) what corresponded with the values of sol fraction and swelling degree.

### 3.4. Thermogravimetric Analysis

**Figure 5:**
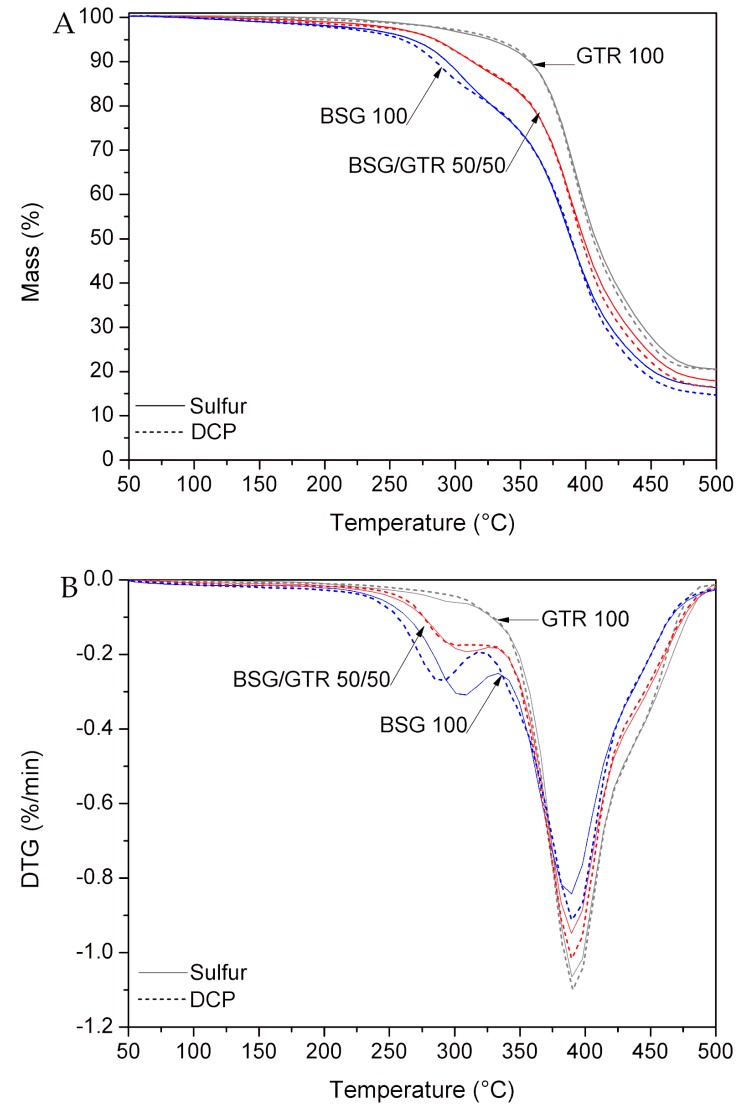
(**A**) TGA and (**B**) DTG curves for NR/BSG/GTR biocomposites.

The results of the thermogravimetric analysis of NR based biocomposites are presented in Figure 5. The degradation of BSG occurs at 309 °C in the system cured with sulfur, and at 285 °C when cured with DCP [47]. The second peak at 390 °C is related to the decomposition of natural rubber. The variation in the size of this signal is related to the content of natural rubber in the samples. Due to the presence of NR in GTR, the peak for samples with a higher amount of GTR, the signal with higher intensity was observed. Furthermore, for GTR and BSG/GTR samples, the third peak appears around 445 °C, which can be associated with the degradation of synthetic rubber present in GTR. Decomposition temperatures of the samples indicate that increasing the content of GTR resulted in the improvement of their thermal stability. This phenomenon is related to the rather lower thermal stability of BSG (lignocellulose waste) in comparison to NR and GTR (polymers/composites). Increasing the content of GTR, the decomposition of final products starts later. Moreover, the char residue is higher for samples containing GTR. This is due to the presence of carbon black and silica in waste tires. For studied biocomposites, the type of curing system has an insignificant impact on the thermal stability and char residue.

### 3.5. Scanning Electron Microscopy

**Figure 6:**
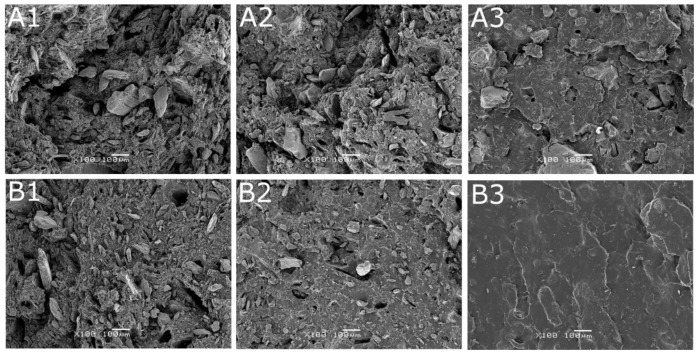
SEM images of samples: (**A1**) NR/BSG100^S^, (**A2**) NR/BSG/GTR50/50^S^, (**A3**) NR/GTR100^S^, (**B1**) NR/BSG100^P^, (**B2**) NR/BSG/GTR50/50^P^, (**B3**) NR/GTR100^P^ (magnification ×100).

**Figure 7:**
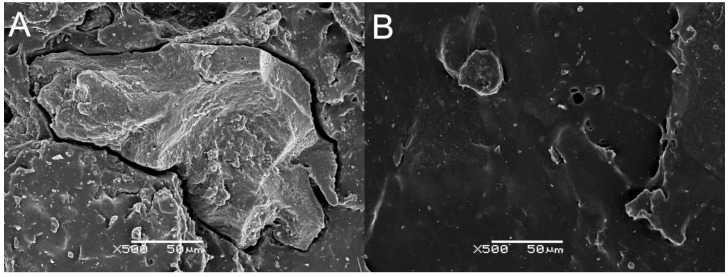
SEM images of samples: (**A**) NR/GTR100^S^, (**B**) NR/GTR100^P^ (magnification ×500).

Micrographs of tensile fractured NR/BSG_100_, NR/BSG/GTR_50/50_, and NR/GTR_100_ (1, 2, and 3, respectively) cured with sulfur-based system (A) and DCP (B) are presented in Figure 6. The structure presented in picture A1 has a rough surface and many gaps, voids, and the BSG filler is poorly dispersed. BSG 100 sample cured with DCP (B1) seems to be smoother and better dispersed. In general, both samples have poor morphological properties, which is due to the nature of applied materials (NR—hydrophobic, BSG—hydrophilic). NR/BSG/GTR_50/50_^S^ and NR/BSG/GTR_50/50_^P^ (A2 and B2) show a more homogenous structure due to the similar nature of NR and GTR. It can be seen that samples with the absence of BSG (A3 and B3) have significantly better surface quality than other samples. It was observed that GTR was well-embedded in the natural rubber matrix when DCP radicals initiator was applied compared to the sulfur curing system. For further analysis of this phenomenon, the micrographs of NR/GTR_100_ cured with sulfur curing system and DCP are presented in higher magnification (×500) in Figure 7. The evident difference in the physical structure of presented samples is caused by the different curing temperatures (160 °C and 180 °C for NR/GTR_100S_ and NR/GTR_100P_, respectively) affecting the efficiency of reclaiming process. Even though the structure of NR/GTR_100_^P^ is smoother than NR/GTR_100_^S^, the mechanical properties (tensile strength, elongation at break, and modulus at 100%) of the sample are lower (Section 3.3). Additionally, in this case, the temperature may have significant influence. It is well-known that NR starts to be degraded at 200 °C, however, the mastication process of studied biocomposites could influence the physical and chemical structure of NR, shifting the degradation temperature to lower values. In that case, the structure of the studied sample look smoother and more homogeneous, however, the thermal degradation process resulted in deterioration of the mechanical performance.

When analyzing samples with BSG, it may be assumed that the BSG filler is better dispersed in samples with DCP. It can be related to the curing efficiency of the studied samples. The highly cross-linked structure prevents BSG from proper matrix penetration, resulting in less homogenous structure and a higher amount of gaps and voids. The structure of the sample with GTR 100 and cured with DCP seems to be much more uniform than samples cured with sulfur. Presented SEM micrographs prove that the structure of the blends depends on the type of curing system.

### 3.6. Acoustic Properties

**Figure 8:**
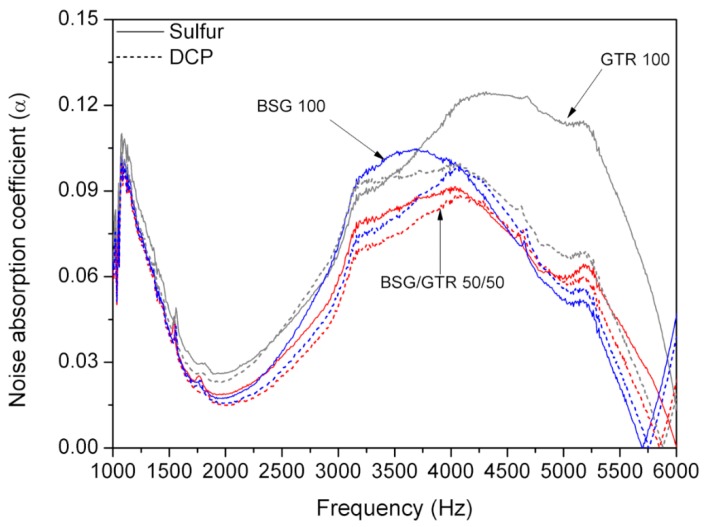
Sound absorption coefficient as a function of frequency for NR/BSG/GTR biocomposites.

The sound absorption coefficients of NR blended with BSG, BSG/GTR, and GTR and cured with different agents are presented in Figure 8. It can be seen that at frequencies near 1000 Hz, all samples characterize with similar sound absorption. This phenomenon is due to the presence of NR in all studied biocomposites. It was observed that samples cured with DCP show a lower sound absorption. The presence of voids and gaps created during sample preparation enhances the acoustic properties of the material [48]. It was confirmed by the morphology of studied samples that the sulfur curing system is more prone to form free spaces in the structure of the samples compared to samples with DCP system.

Figure 8 shows that in the region of high frequencies (from 3000 to 6000 Hz), samples with GTR 100 absorb in a larger range than samples with BSG 100 as a filler. That means that the sulfur curing system produced a higher absorption noise coefficient than the DCP curing system. It seems that this difference is due to cross-linking efficiency provided by the sulfur system when GTR is present. The cross-linked structure can also act avoiding the collapse of the voids and occlusions, this effect would be lower in the case of DCP.

## 4. Conclusions

In this paper, natural rubber-based materials filled with BSG, GTR, and BSG/GTR were prepared in a Brabender batch mixer followed by cross-linking with sulfur curing system or dicumyl peroxide. The impact of fillers and curing system on obtained samples was evaluated by the analysis of curing characteristics, chemical structure, physico-mechanical, thermal, morphological, and acoustic properties.

Curing parameters indicate that the sulfur curing system is more sensitive to fillers change compared to DCP in terms of processing, while maximal torque and difference between minimal and maximal torque keep the same tendency. This phenomenon may indicate that curing using DCP is not affected by the migrating components of GTR filler. The micrographs have shown that the tensile fracture surface of samples was more homogenous for NR/GTR_100_^P^. Conducted studies show that the type of curing agent has a significant influence on curing, mechanical, and physical properties, mainly by cross-linking efficiency. Samples cured with the sulfur-based system show better mechanical properties than samples with DCP, due to better compatibility between NR and GTR particles than BSG. Additionally, the chain restriction is smaller for samples containing a higher amount of GTR, where a high quantity of BSG caused limitation of polymer chains mobility. Moreover, the type of filler used also affects the effectiveness of the applied curing additives, for instance by its partial absorption or different affinity to applied waste fillers and the natural rubber matrix. During the rheological analysis, it was shown that the higher the ratio of BSG, the higher M_H_ and the lower M_L_ values are. The dual nature of BSG acts improves processing, but in later stages of vulcanization immobilizes the polymer chains. The opposite phenomenon occurs for GTR. The vulcanized particles hinder the processing, but due to the high temperature, eventual reclaiming occurs (on the surface of GTR particles), improving the compatibility with NR matrix (confirmed by SEM). The mechanical properties show that the application of BSG does not influence positively the strain-induced crystallization, hindering the formation of a semi-crystal structure.

## Figures and Tables

**Table 1:** Characteristics of used components.

Component	Abbreviation	Function	Characteristics	Company	Treatment before Use
Natural rubber	NR	matrix	Ribbed Smoked Sheets type with density 0.92 g/cm^3^	Guma-Pomorska (Głobino, Poland)	Used as received.
Ground tire rubber	GTR	filler	Particles size: below 0.8 mm	Orzeł S.A. (Poniatowa, Poland)	Prior to processing, GTR was mechano-chemically reclaimed at ambient temperature using two-roll mills. GTR in presence of bitumen was masticated for 10 min (after this time homogenous material was formed), in order to enhance matrix-filler interactions [36,37].
Brewer’s spent grain	BSG	filler	Initial composition: wheat malts 47.7 wt.% and barley malts 52.3 wt.% Particles size: below 0.8 mm	Dno Bojlera (Rotmanka, Poland)	Prior to processing, BSG was dried at 80 °C and then mechanically grounded in a co-rotating twin-screw extruder at 120 °C in order to obtain particles with a similar size distribution.
*N*-tert-butyl-2-benzothiazole sulfonamide	TBBS	accelerator	Molar mass: 238.37 g/mol Density: 1.29 g/cm^3^ Melting temperature: 104–111 °C	Standard Sp. z o.o. (Lublin, Poland)	Used as received.
tetramethylthiuram disulfide	TMTD	accelerator	Molar mass: 240.43 g/mol Density: 1.38 g/cm^3^ Melting temperature: 146–148 °C	Standard Sp. z o.o. (Lublin, Poland)	Used as received.
Stearic acid	St	plasticizer/activator	Molar mass: 284.48 g/mol Density: 0.94 g/cm^3^ Melting temperature: 69.3 °C	Standard Sp. z o.o. (Lublin, Poland)	Used as received.
Zinc oxide	ZnO	activator	Molar mass: 81.38 g/mol Density: 5.61 g/cm^3^	Standard Sp. z o.o. (Lublin, Poland)	Used as received.
Sulfur	S	radicals initiator	Density: 2.07 g/cm^3^ Melting temperature: 115 °C	Standard Sp. z o.o. (Lublin, Poland)	Used as received.
Dicumyl peroxide	DCP	radicals initiator	Molar mass: 270.37 g/mol Density: 1.56 g/cm^3^ Melting temperature: 39–41 °C	Pergan GmbH (Bocholt, Germany)	Used as received.

**Table 2:** Composition and curing characteristics of studied biocomposites.

Components (phr)	NR/BSG_100_^S^	NR/BSG/GTR_50/50_^S^	NR/GTR_100_^S^	NR/BSG_100_^P^	NR/BSG/GTR_50/50_^P^	NR/GTR_100_^P^
NR	100	100	100	100	100	100
GTR	0	50	100	0	50	100
BSG	100	50	0	100	50	0
Curing characteristics at 160 °C (sulfur system)				Curing characteristics at 180 °C (DCP)		
Minimal torque (dNm)	1.7	5.0	4.8	1.9	1.8	3.8
Maximal torque (dNm)	41.3	30.9	23.6	45.9	28.6	20.0
ΔM (dNm)	39.6	25.9	18.8	44.0	26.8	16.2
Scorch time (t_2_, min)	3.9	2.0	2.6	1.3	1.3	1.3
Optimum cure time (t_90_, min)	10.1	6.4	5.7	4.1	4.1	4.1
Cure rate index (CRI. Min^−1^)	16.1	22.5	31.8	34.6	35.5	35.6
Thermal aging resistance (R_300_%)	0.5	0.6	1.1	1.6	1.3	1.9

S—sulfur curing system (in phr): zinc oxide 5.0; stearic acid 3.0; TBBS 1.0; TMTD 0.25; sulfur 2.0. P—dicumyl peroxide 3.0 phr.

**Table 3:** Physico-mechanical properties of NR-based biocomposites.

Components (phr)	NR/BSG_100_^S^	NR/BSG/GTR_50/50_^S^	NR/GTR_100_^S^	NR/BSG_100_^P^	NR/BSG/GTR_50/50_^P^	NR/GTR_100_^P^
NR	100	100	100	100	100	100
GTR	0	50	100	0	50	100
BSG	100	50	0	100	50	0
Physico-mechanical properties						
Tensile strength (MPa)	4.5 ± 0.1	6.2 ± 1.4	8.0 ± 0.4	2.7 ± 0.1	3.9 ± 0.7	3.3 ± 0.7
Elongation at break (%)	475 ± 12	452 ± 84	511 ± 27	221 ± 48	341 ± 56	302 ± 78
M100 (MPa)	1.8 ± 0.1	1.5 ± 0.2	0.9 ± 0.1	2.1 ± 0.1	1.2 ± 0.1	0.8 ± 0.1
Hardness (Sh A)	66 ± 1	53 ± 1	44 ± 1	63 ± 1	48 ± 1	35 ± 1
Density at 25 °C (g/cm^3^)	1.11 ± 0.01	1.08 ± 0.01	1.04 ± 0.01	1.09 ± 0.02	1.06 ± 0.01	1.02 ± 0.01
Sol fraction (%)	3.38 ± 0.20	5.34 ± 0.20	7.58 ± 0.24	4.52 ± 0.03	6.58 ± 0.20	8.76 ± 0.19
Swelling degree (%)	232 ± 2	228 ± 6	277 ± 4	141 ± 2	232 ± 4	307 ± 2
Cross-link density (mol/cm^3^ × 10^−4^)	1.80 ± 0.03	0.87 ± 0.02	0.62 ± 0.01	1.89 ± 0.04	0.85 ± 0.03	0.53 ± 0.03

S—sulfur curing system (in phr): zinc oxide 5.0; stearic acid 3.0; TBBS 1.0; TMTD 0.25; sulfur 2.0. P—dicumyl peroxide 3.0 phr.
